# Target of Rapamycin Regulates Photosynthesis and Cell Growth in *Auxenochlorella pyrenoidosa*

**DOI:** 10.3390/ijms231911309

**Published:** 2022-09-25

**Authors:** Tingting Zhu, Linxuan Li, Huimin Chang, Jiasui Zhan, Maozhi Ren

**Affiliations:** 1 Institute of Urban Agriculture, Chinese Academy of Agricultural Sciences, Chengdu National Agricultural Science and Technology Center, Chengdu 610213, China; 2 Hainan Yazhou Bay Seed Laboratory, Sanya 572025, China; 3 School of Agricultural Science, Zhengzhou University, Zhengzhou 450001, China; 4 Department of Forest Mycology and Plant Pathology, Swedish University of Agricultural Sciences, 75007 Uppsala, Sweden

**Keywords:** TOR, photosynthesis, cell growth, AZD8055, *Auxenochlorella pyrenoidosa*

## Abstract

*Auxenochlorella pyrenoidosa* is an efficient photosynthetic microalga with autotrophic growth and reproduction, which has the advantages of rich nutrition and high protein content. Target of rapamycin (TOR) is a conserved protein kinase in eukaryotes both structurally and functionally, but little is known about the TOR signalling in *Auxenochlorella pyrenoidosa*. Here, we found a conserved ApTOR protein in *Auxenochlorella pyrenoidosa*, and the key components of TOR complex 1 (TORC1) were present, while the components RICTOR and SIN1 of the TORC2 were absent in *Auxenochlorella pyrenoidosa*. Drug sensitivity experiments showed that AZD8055 could effectively inhibit the growth of *Auxenochlorella pyrenoidosa*, whereas rapamycin, Torin1 and KU0063794 had no obvious effect on the growth of *Auxenochlorella pyrenoidosa* *a*. Transcriptome data results indicated that *Auxenochlorella pyrenoidosa* TOR (ApTOR) regulates various intracellular metabolism and signaling pathways in *Auxenochlorella pyrenoidosa*. Most genes related to chloroplast development and photosynthesis were significantly down-regulated under ApTOR inhibition by AZD8055. In addition, ApTOR was involved in regulating protein synthesis and catabolism by multiple metabolic pathways in *Auxenochlorella pyrenoidosa*. Importantly, the inhibition of ApTOR by AZD8055 disrupted the normal carbon and nitrogen metabolism, protein and fatty acid metabolism, and TCA cycle of *Auxenochlorella pyrenoidosa* cells, thus inhibiting the growth of *Auxenochlorella pyrenoidosa*. These RNA-seq results indicated that ApTOR plays important roles in photosynthesis, intracellular metabolism and cell growth, and provided some insights into the function of ApTOR in *Auxenochlorella pyrenoidosa*.

## 1. Introduction

*Chlorella* is a unicellular eukaryotic green alga that emerged 2 billion years ago and is a high-efficiency primary producer in ecosystems [1]. *Chlorella* can be cultured in a natural environment or in controllable closed systems, with higher productivity than most plants. For a long time, *Chlorella* has been deemed as a source of protein and fat, and it is used as a raw material for human food and animal feed [2,3]. Like land plants, *Chlorella* also performs photosynthesis via chloroplast, converting solar energy into chemical energy that is vital to its development and generates oxygen. *Chlorella* contains many high-value substances such as protein, pigment, antioxidants, vitamins, minerals and cell growth factor, and has been referred to as “the best genetic food in the 21st century” by the World Health Organization [4]. At present, over 10 species of *Chlorella* have been identified in the world [5,6], among which *Auxenochlorella pyrenoidosa* (*A. pyrenoidosa*, formerly *Chlorella pyrenoidosa*) has attracted much attention because it is edible and its protein content can account for more than 50% of dry weight [7]. The genome size of *A. pyrenoidosa* FACHB-9 is 56.6 Mbp, including 10,284 genes [8]. An analysis of the genome structure provides a foundation for improving *A. pyrenoidosa* production as food and fuels. Furthermore, *A. pyrenoidosa* has been widely used in wastewater treatment, especially in high-concentration inorganic industrial wastewater [9]. Utilizing high ammonium salts in industrial wastewater can produce up to 56.7% (dry weight) protein in *A. pyrenoidosa*, and 95% of ammonium salt could be consumed [10]. Additionally, *A. pyrenoidosa* also utilizes organic carbon source and nitrogen source for high-density heterotrophic growth, with production efficiency being over ten times higher than that of autotrophic growth [8]. However, chloroplast was degraded, lipid content was increased, and protein synthesis was inhibited in heterotrophic *A. pyrenoidosa* [8,11,12]. Genomic and transcriptomic sequencing results showed that heterotrophic to photoautotrophic of *A. pyrenoidosa* resulted in global metabolic reprogramming [8].

Target of rapamycin (TOR) is a core regulatory factor for eukaryotic growth and development, which coordinates cell proliferation, growth and metabolism [13,14]. TOR protein has highly conserved structures, including N-terminal HEAT repeats, FAT, FRB, catalytic and C-terminal FATC domains [15]. TOR protein and other proteins form TOR complex 1 (TORC1) and TORC2 in yeast and mammals [16,17]. However, there was only the conserved and functional TORC1 in plants, and TORC1 was composed of TOR, regulatory associate the protein target of rapamycin (RAPTOR) and lethal with sec-13 protein 8 (LST8) [18,19]. TORC2 core proteins RICTOR and SIN1 seem to be missing in photosynthetic eukaryotes, including plants and green algae [20,21].

Rapamycin is a macrolide immunosuppressant from the bacterium *Streptomyces hygroscopicus*. It binds to the 12 kDa FK506 binding protein (FKBP12) and the FRB domain of TOR, thereby inhibiting the activity of TOR protein [22]. Loss of FKBP12 function prevents rapamycin from inhibiting the TOR protein in most plants [23,24,25,26]. Fortunately, TOR kinase inhibitors such as Torin1, AZD8055 and KU0063794 from mammals were developed and applied in plants, and have also been proved to specifically and efficiently inhibit TOR kinase activity in plants [15,27,28]. With the help of TOR inhibitors and various omics research methods, animal and plant conserved and plant-specific TOR signaling pathways have been revealed [18,29]. In plants, TOR regulates cell division and elongation, protein synthesis, nutrient and metabolism, and stress response by integrating multiple exogenous environmental signals and endogenous physiological signals [15,30,31,32]. TOR affects plant growth and development from embryogenesis to photomorphogenesis, root and leaf development, flowering, and senescence in plants [13,18,33].

Genomic analysis of some algal species revealed that TORC1 components are widely conserved in algae [34]. Different from other microalgae, the functions of TOR have been comprehensively studied in the model green alga *Chlamydomonas reinhardtii* (*C. reinhardtii*) [35]. Previous studies have shown that *C. reinhardtii* is sensitive to rapamycin, and the rapamycin sensitive TORC1 signaling regulates cell growth, protein synthesis, autophagy, and key metabolism processes in *C. reinhardtii* [35,36,37]. In addition, a recent study has shown that TOR controls the carotenoid production by phosphorylating lycopene beta/epsilon cyclase in *C. reinhardtii* [38]. This is the first evidence that TOR directly regulates the biosynthesis of secondary metabolite carotenoid in algae. As an industrial production alga, *A. pyrenoidosa* has fast growth rate, rich nutrition and high protein. However, the TOR signaling pathway of *A. pyrenoidosa* has not been reported, and whether TOR signaling regulates the cell growth and protein synthesis of *A. pyrenoidosa* remains unknown. In this study, homologous sequence alignment revealed that there was only the conserved TORC1 signaling pathway in *A. pyrenoidosa*. Drug sensitivity experiments showed that AZD8055 could effectively inhibit the growth of *A. pyrenoidosa*, while rapamycin, Torin1 and KU0063794 had no effect on the growth of *A. pyrenoidosa*. RNA-seq results showed that most genes involved in photosynthesis were significantly down-regulated in *A. pyrenoidosa* treated with AZD8055, indicating that ApTOR had an important effect on photosynthesis of *A. pyrenoidosa*. In addition, DEGs involved in the regulation of autophagy and ubiquitin mediated proteolysis were almost all up-regulated, suggesting that ApTOR was also involved in regulating autophagy and protein catabolic process of *A. pyrenoidosa*. These results suggested that ApTOR plays major roles in regulating photosynthesis and cellular metabolism in *A. pyrenoidosa*.

## 2. Results

### 2.1. Conserved TOR Signaling Pathway in Auxenochlorella Pyrenoidosa

**Figure 1:**
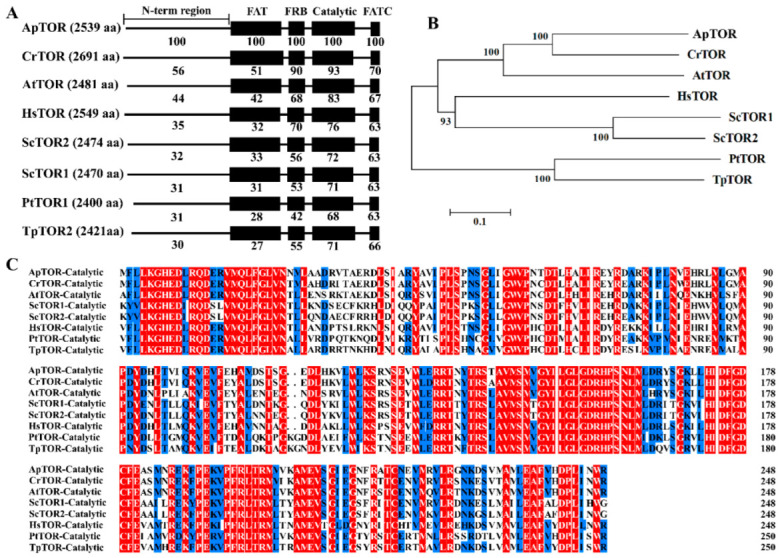
A structure and sequence analysis of ApTOR. (**A**) An analysis of the conserved domains of ApTOR protein and homologs from other species. The number denotes the identity (%) of ApTOR domain with homologs from other species. *Chlamydomonas reinhardtii* (Cr) (Chlorophyta), *Arabidopsis thaliana* (At) (Plantae, Magnoliophyta), *Homo sapiens* (Hs) (Animalia, Chordata), *Saccharomyces cerevisiae* (Sc) (Fungi, Ascomycota), *Phaeodactylum tricornutum* (Pt) (Bacillariophyta), *Thalassiosira pseudonana* (Tp) (Bacillariophyta). (**B**) The phylogenetic tree of ApTOR protein and homologs from other species. The phylogenetic tree was constructed by MEGA 4 software using the Neighbor-Joining method. Numbers represent bootstrap percentages (1000 of bootstrap replicates). (**C**) Sequence alignment of the catalytic domains of ApTOR protein and homologs from other species. Red represents identical amino acid sequences, and blue represents more than 75% identical amino acid sequences.

The conserved TORC1 signal regulates intracellular metabolism, nutrient, and energy in *C. reinhardtii* [35,36,37]. To investigate the conserved TOR signaling pathway in *A. pyrenoidosa*, BLASTp analysis was performed on the public transcriptome data of *A. pyrenoidosa* (NCBI accession number: PRJNA730327). Only one conserved TOR protein (ApTOR) was found in *A. pyrenoidosa*, with a maximum similarity of 69% to CrTOR protein. ApTOR contains N-terminal HEAT repeats, FAT, FRB, catalytic and FATC domains at C-terminal (Figure 1A). Homologous sequence alignment revealed that the catalytic domain of ApTOR was the most conserved with the highest similarity among species, while the FAT domain had the lowest similarity among species (Figure 1A,C). Phylogenetic tree showed that ApTOR and CrTOR were the most conserved in evolution but had the most distant evolutionary relationship with TpTOR and PtTOR (Figure 1B). Meanwhile, sequence alignment found that RAPTOR and LST8, the key proteins of TORC1, were also present in *A. pyrenoidosa* (Table 1), whereas RICTOR and SIN1, the key proteins of TORC2, were not found in *A. pyrenoidosa*. In addition, TORC1 signaling downstream components also existed in *A. pyrenoidosa* (Table 1). These results showed that there was a conserved TORC1 signaling pathway in *A. pyrenoidosa*.

### 2.2. Effects of TOR Inhibitors on the Growth of Auxenochlorella Pyrenoidosa

**Figure 2:**
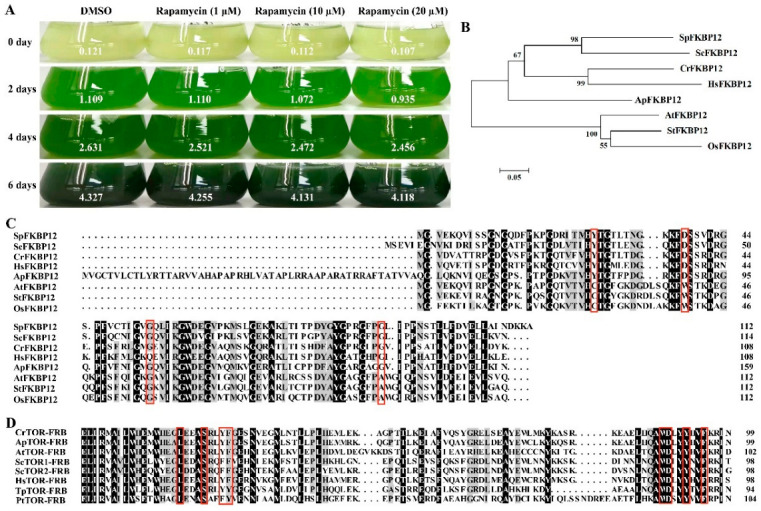
*Auxenochlorella pyrenoidosa* is resistant to rapamycin. (**A**) The phenotype of *A. pyrenoidosa* treated with different concentrations of rapamycin for 0, 2, 4, and 6 days. The numbers denote the corresponding OD680nm values. (**B**) The phylogenetic tree of ApFKBP12 protein and homologs from other species. Phylogenetic tree was constructed by MEGA 4 software using the Neighbor-Joining method. Numbers represent bootstrap percentages (1000 of bootstrap replicates). *Schizosaccharomyces pombe* (Sp) (Fungi, Ascomycota), *Oryza sativa* (Os) (Plantae, Tracheophyta), *Solanum tuberosum* (St) (Plantae, Tracheophyta). (**C**) Sequence alignment of the ApFKBP12 protein and homologs from other species. The red rectangle denotes the amino acid required for FKBP12 binding to rapamycin. (**D**) Sequence alignment of the FRB domains of the ApTOR protein and homologs from other species. The red rectangle denotes the amino acid required for the FRB domain binding to rapamycin.

In order to elucidate function of TOR signal in *A. pyrenoidosa*, *A. pyrenoidosa* was treated with rapamycin, a specific inhibitor of TOR protein. The results showed that rapamycin has no obvious effect on the growth of *A. pyrenoidosa*, even at a higher concentration of rapamycin (Figure 2A), indicating that *A. pyrenoidosa* is insensitive to rapamycin. Previous studies have shown that rapamycin inhibits the TOR activity by forming a ternary complex with FKBP12 and the FRB domain of TOR [39]. A ApFKBP12 sequence with 43% similarity to CrFKBP12 was found in the transcriptome data of *A. pyrenoidosa*, encoding 159 amino acids (Table 1). Interestingly, ApFKBP12 protein is evolutionarily closer to rapamycin-sensitive species (Figure 2B). Some amino acids involved in the formation of the FKBP12-rapamycin complex are required for inhibiting TOR activity [40]. We found that the ApFKBP12 protein sequence contains the conserved amino acids required for FKBP12 binding to rapamycin, including Tyr26, Asp38 and Gly89 (numbered according to human HsFKBP12) (Figure 2C). However, there was an additional sequence of 52 amino acids at the N-terminal of ApFKBP12 protein compared with other species (Figure 2C), which may change the function of ApFKBP12 protein and failure in binding to rapamycin. Rapamycin also interacts with the FRB domain of TOR by binding to aromatic residues [40], and sequence alignment revealed that these key amino acids were highly conserved in *A. pyrenoidosa* and other species (Figure 2D). As the FRB domain of ApTOR is highly conserved, the resistance of *A. pyrenoidosa* to rapamycin may be due to the loss of ApFKBP12 function.

**Figure 3:**
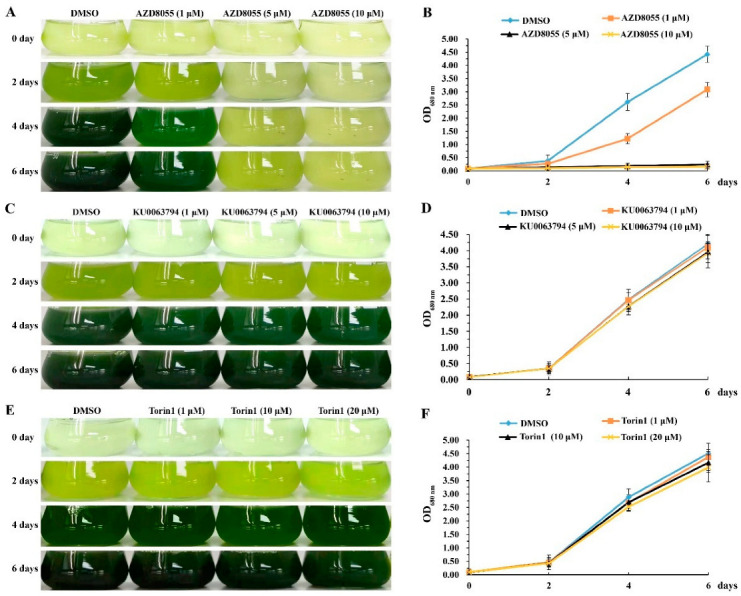
The effects of TOR protein inhibitors on the growth of *Auxenochlorella pyrenoidosa.* (**A**) AZD8055 inhibits the growth of *A. pyrenoidosa* in a dose-dependent manner. (**B**) Change curves of OD680nm values of *A. pyrenoidosa* treated with 1, 5 and 10 μM AZD8055 for 0, 2, 4 and 6 days. (**C**) Phenotype of *A. pyrenoidosa* treated with 1, 5 and 10 μM KU0063794 for 0, 2, 4, and 6 days. (**D**) Change curves of OD680nm values as described in (**C**). (**E**) Phenotype of *A. pyrenoidosa* treated with 1, 10, 20 μM Torin1 for 0, 2, 4, and 6 days. (**F**) Change curves of OD680nm values as described in (**E**).

Furthermore, TOR kinase inhibitors AZD8055, Torin1 and KU0063794 were used to treat *A. pyrenoidosa*. The results showed that AZD8055 could effectively inhibit the growth of *A. pyrenoidosa*, while Torin1 and KU0063794 had no effect on the growth of *A. pyrenoidosa* even at higher concentrations (Figure 3). The 50% inhibitory concentration (IC50) of AZD8055 on the growth of *A. pyrenoidosa* was about 1 μM. When the concentration of AZD8055 reached 5 μM, the growth of *A. pyrenoidosa* was completely inhibited, indicating that the lethal concentration of AZD8055 may be 5 μM (Figure 3A,B). However, when AZD8055 was removed from the medium, the inhibited cells resumed growth (Supplementary Figure S1), indicating that inhibition of ApTOR kinase activity by AZD8055 prevents cell division without killing cells. These results suggested that AZD8055 can be applied to elucidate the function of ApTOR in *A. pyrenoidosa*.

### 2.3. Analysis of Transcriptome Sequencing under ApTOR Inhibition

**Figure 4:**
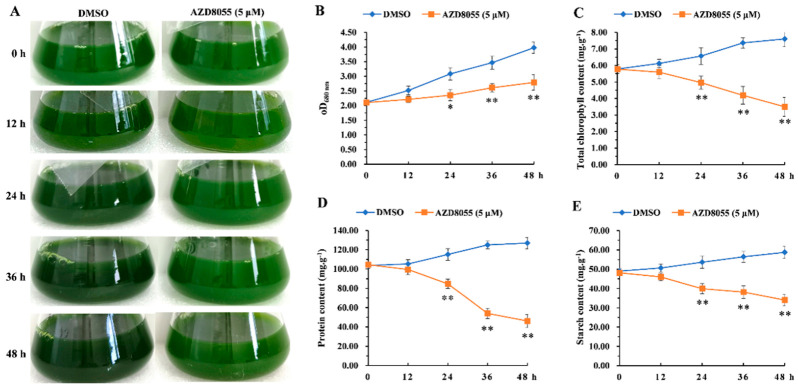
TOR regulates the biosynthesis of major intracellular substances in *Auxenochlorella pyrenoidosa*. (**A**) Phenotypes of *A. pyrenoidosa* treated with AZD8055 for 0, 12, 24, 36, and 48 h. *A. a pyrenoidosa* was cultured in 50 mL BG11 liquid medium for 4 days. Then, final-concentration 5 μM AZD8055 or equivalent DMSO was added into the alga solution for 0, 12, 24, 36, and 48 h. (**B**) Change curves of OD680nm values of *A. pyrenoidosa* treated with 5 μM AZD8055 for 0, 12, 24, 36, and 48 h. (**C**) Total chlorophyll content of *A. pyrenoidosa* treated with 5 μM AZD8055 for 0, 12, 24, 36, and 48 h. (**D**) Protein content of *A. pyrenoidosa* treated with 5 μM AZD8055 for 0, 12, 24, 36, and 48 h. (**E**) Starch content of *A. pyrenoidosa* treated with 5 μM AZD8055 for 0, 12, 24, 36, and 48 h. Fresh weight of *A. pyrenoidosa* was used to measure chlorophyll, protein and starch contents, respectively. The data represents the mean ± SD of n = 3 independent experiments. Asterisks denote Student’s *t*-test significant difference compared with DMSO (* *p* < 0.05; ** *p* < 0.01).

To further clarify the roles of ApTOR signaling pathway in *A. pyrenoidosa*, the transcriptome sequencing was performed in *A. pyrenoidosa* treated with AZD8055. The growth curve showed that *A. pyrenoidosa* was in the logarithmic phase after incubation for 4 days (Figure 3); we therefore cultured the algal cells for 4 days before AZD8055 treatment. In addition, we found that the OD_680nm_ value, chlorophyll content, protein content, and starch content of *A. pyrenoidosa* were significantly changed during *A. pyrenoidosa* treated with 5 μM AZD8055 for 24 h (Figure 4); thus, *A. pyrenoidosa* treated with 5 μM AZD8055 for 24 h was used for transcriptome sequencing.

**Figure 5:**
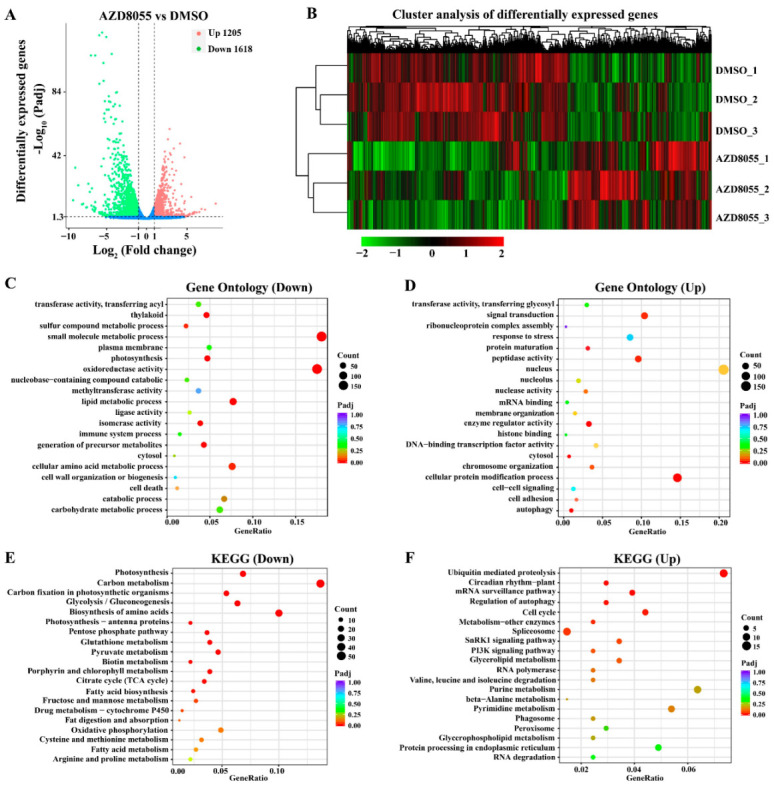
The transcriptome data analysis of AZD8055-treated *Auxenochlorella pyrenoidosa*. (**A**) Number of up- and down-regulated DEGs between AZD8055 and DMSO treatment. (**B**) Cluster analysis of DEGs between AZD8055 and DMSO treatment. The color represents the FPKM value of the gene by Z-score. Red denotes high gene expression and green denotes low gene expression. (**C**) The top 20 enriched gene ontology in down-regulated DEGs. (**D**) The top 20 enriched gene ontology in up-regulated DEGs. (**E**) The top 20 enriched KEGG pathways in down-regulated DEGs. (**F**) The top 20 enriched KEGG pathways in up-regulated DEGs.

*A. pyrenoidosa* was cultured for 4 days, and then final-concentration 5 μM AZD8055 or equivalent DMSO was added into the algal solution for 24 h. Subsequently, AZD8055-treated algal cells were used for transcriptome sequencing. After filtering the raw data, clean reads for subsequent analysis were obtained, and the data summary is as shown in Supplementary Table S1. A total of 2823 differentially expressed genes (DEGs) were found between AZD8055 treatment and DMSO control, of which 1205 DEGs were up-regulated and 1618 were down-regulated (Figure 5A). To verify the reliability of transcriptome data, 10 DEGs were randomly selected from the transcriptome data for qRT-PCR. The qRT-PCR results showed the same trend as the transcriptome data (Supplementary Figure S2), indicating that the transcriptome data were valid and reliable.

A cluster analysis of DEGs showed that the transcription levels of many genes were changed in *A. pyrenoidosa* treated with AZD8055 compared to the DMSO control (Figure 5B). To clarify the functions of DEGs, GO functional enrichment analysis was conducted, and a total of 121 down-regulated GO terms and 124 up-regulated GO terms were enriched in the transcriptome data. Among the down-regulated GO terms, thylakoid (GO:0009579) and photosynthesis (GO:0015979) were most significant enrichment (Figure 5C). Among the up-regulated GO terms, the cellular protein modification process (GO:0006464) and autophagy (GO:0006914) were most significant enrichment (Figure 5D). These results suggested that ApTOR regulates multiple biological processes in *A. pyrenoidosa*. A KEGG pathway enrichment analysis showed that photosynthesis, carbon metabolism and carbon fixation in photosynthetic organisms were most significant enrichment in the down-regulated DEGs (Figure 5E). Among the up-regulated KEGG pathways, ubiquitin mediated proteolysis and circadian rhythm-plant were most significantly enriched (Figure 5F). These results suggested that ApTOR controls various intracellular metabolism and signaling pathways in *A. pyrenoidosa*.

### 2.4. DEGs Involved in Regulating Chloroplast Development and Photosynthesis of Auxenochlorella Pyrenoidosa

Chloroplasts containing chlorophyll are necessary for photosynthesis in plants [41]. Previous studies showed that TOR has the function of regulating chloroplast development and photosynthesis in *Arabidopsis* [42]. Down-regulated GO terms related to photosynthesis and chloroplast development were enriched in the transcriptome data (Figure 5C). Meanwhile, metabolic pathways related to plant photosynthesis were also found in the KEGG pathways, such as photosynthesis, carbon fixation in photosynthetic organisms, and porphyrin and chlorophyll metabolism (Figure 5E). These results indicated that ApTOR has important effects on chloroplast development and photosynthesis in *A. pyrenoidosa*.

A total of 62 DEGs were associated with photosynthesis under ApTOR inhibition, among which 29, 9 and 24 DEGs were enriched in the “Photosynthesis”, “Photosynthesis-antenna proteins” and “Carbon fixation in photosynthetic organisms” KEGG pathways, respectively (Table 2). Most DEGs related to photosynthesis were significantly down-regulated, and all DEGs of photosystem I, photosystem II and chlorophyll a/b binding protein pathways were down-regulated under ApTOR inhibition (Table 2 and Supplementary Figure S3). The highest down-regulated gene was *Chlorophyll a-b binding protein 4* (*Cluster-495.6691*) gene with 131.60-fold decrease. In the dark reaction of photosynthesis, 22 down-regulated DEGs and 2 up-regulated DEGs were involved in carbon fixation. In addition, all 16 chlorophyll synthesis genes involved in “Porphyrin and chlorophyll biosynthesis” pathway were down-regulated from 2.27- to 27.67-fold, and 44 down-regulated DEGs and 6 up-regulated DEGs were involved in the “Thylakoid” pathway (Supplementary Table S2). These results suggested that ApTOR positively regulates chloroplast development and photosynthesis in *A. pyrenoidosa*.

### 2.5. DEGs Involved in Regulating Protein Synthesis and Catabolism of Auxenochlorella Pyrenoidosa

*A. pyrenoidosa* has a high protein content, but whether ApTOR regulates the protein synthesis of *A. pyrenoidosa* remains unknown. Previous studies have shown that ribosomes are responsible for protein synthesis in all organisms, and TOR plays an essential role in regulating ribosome synthesis [43,44,45]. In this study, genes involved in the “Ribosome biogenesis” pathway were significantly changed in *A. pyrenoidosa*, including ribosomal proteins and U3 small nucleolar ribonucleoprotein proteins. A total of 40 DEGs were enriched in the KEGG “Ribosome biogenesis” pathway, including 26 down-regulated DEGs and 14 up-regulated DEGs, and most of DEGs were ribosome proteins (Supplementary Table S3). Importantly, most of DEGs associated with ribosomal proteins were significantly down-regulated, and the most down-regulated gene was *50S ribosomal protein L2* (*Cluster-829.0*) with 26.17-fold decrease (Supplementary Table S3). These results indicated that ApTOR inhibition leads to dysfunction of ribosomes, especially changes in ribosomal protein-related genes, further indicating that ApTOR controls protein synthesis by ribosomes.

Autophagy plays a central role in protein degradation, and previous studies showed that TOR negatively regulates autophagy [46,47,48]. In this study, transcriptome analysis showed that autophagy related DEGs were significantly enriched in GO terms and KEGG pathways (Figure 5D,F). Total 8 DEGs were assigned to the “Regulation of autophagy” pathway, of which 7 genes were up-regulated including *SnRK1α* and *ATG* genes, and 1 gene was down-regulated in the RNA-seq data (Table 3). These results suggested that ApTOR negatively regulates autophagy in *A. pyrenoidosa*. Ubiquitin (Ub)/26S proteasome system (UPS) is the main pathway of protein degradation in cells. Ub is sequentially covalently linked to the target protein by ubiquitin activase (E1), ubiquitin binding enzyme (E2), and ubiquitin protein ligase (E3), and then the target protein is degraded by the proteasome [49,50]. The “Ubiquitin mediated proteolysis” KEGG pathway was influenced by AZD8055 (Figure 5F). Total 19 DEGs were assigned to the “Ubiquitin mediated proteolysis” pathway, including 15 up-regulated genes and 4 down-regulated genes (Table 3). Four genes encoding E1 activating enzyme were up-regulated from 2.28- to 7.84-fold under ApTOR inhibition. In addition, some important E3 ubiquitin ligase genes, including *Cullin 1*, *Cullin 3*, and *Cullin 4*, were significantly up-regulated (Table 3). These results showed that ApTOR inhibition activated protein catabolism in *A. pyrenoidosa*.

### 2.6. DEGs Involved in Regulating the Cell Growth of Auxenochlorella Pyrenoidosa

Carbon and nitrogen metabolism, protein and fat synthesis are important limiting factors of cell growth and proliferation [51,52]. In this study, the genes associated with carbon metabolism, amino acid metabolism and fatty acid metabolism were significantly changed under ApTOR inhibition (Supplementary Table S4). DEGs of carbon metabolism and biosynthesis of amino acids and fatty acid pathways were significantly enriched in the down-regulated KEGG pathways (Figure 5E). A total 65 DEGs were assigned to the “carbon metabolism” pathway, including 56 down-regulated genes and 9 up-regulated genes. Some rate-limiting enzyme genes in the “carbon metabolism” pathway such as fructose bisphosphate aldolase and pyruvate kinase were significantly down-regulated. A total 45 DEGs were assigned to the “biosynthesis of amino acids” pathway, including 41 down-regulated genes and 4 up-regulated genes. In addition, all 10 DEGs assigned to the “fatty acid biosynthesis” pathway were down-regulated from 2.53- to 28.44-fold (Supplementary Table S4), indicating that AZD8055 inhibited the biosynthesis of fatty acids in *A. pyrenoidosa*. These results suggested that ApTOR inhibition affects a variety of intracellular metabolic processes, especially carbon and nitrogen metabolism and fatty acid metabolism. The disruption of metabolic homeostasis by AZD8055 may help to inhibit the growth of *A. pyrenoidosa* cells. Consistent with the growth phenotype of *A. pyrenoidosa* treated with AZD8055, all 14 DEGs related to tricarboxylic acid (TCA) cycle were down-regulated from 2.18- to 11.98-fold in the transcriptome data, including rate-limiting enzymes isocitrate dehydrogenase, α-oxoglutarate dehydrogenase and pyruvate dehydrogenase (Table 4), implying that AZD8055 inhibited cell growth of *A. pyrenoidosa* by inhibiting TCA cycle and reducing energy supply.

## 3. Discussion

TOR regulates protein synthesis, intracellular metabolism and cell proliferation by integrating nutrients, energy and environmental signals [13,14,53]. In this study, we provide some new insights into how ApTOR controls multiple cellular processes to regulate cell growth of *A. pyrenoidosa*. Only TORC1 is found in higher plants and the green algae *C. reinhardtii*, which contains key proteins TOR, RAPTOR and LST8. TORC1 activity is regulated by nutrients and environmental stresses and responds to different environmental conditions by controlling intracellular metabolic processes [21,35]. Consistent with the results of higher plants and *C. reinhardtii*, only one conserved ApTOR protein was found in *A. pyrenoidosa* (Figure 1 and Table 1). The key components RAPTOR and LST8 of TORC1 were present, while the components RICTOR and SIN1 of the TORC2 were absent in *A. pyrenoidosa*, implying that the conserved TORC1 pathway exists in *A. pyrenoidosa*.

Studies have shown that *C. reinhardtii* is sensitive to rapamycin [54]. Unexpectedly, we found that rapamycin had no obvious effect on the growth of *A. pyrenoidosa*, even at a higher concentration of rapamycin (20 μM) (Figure 2), showing that *A. pyrenoidosa* is insensitive to rapamycin. Phylogenetic tree analysis and amino acid sequence alignment showed that the resistance of *A. pyrenoidosa* to rapamycin may be caused by the loss of ApFKBP12 function. In addition, we found that AZD8055 could effectively inhibit the growth of *A. pyrenoidosa*, while Torin1 and KU0063794 had no effect on the growth of *A. pyrenoidosa* even at higher concentrations, implying that Torin1 and KU0063794 could not act on the kinase domain of ApTOR protein due to amino acid variation.

Photosynthesis is a plant-specific physiological activity, providing energy and sugars for plants autotrophic growth, which is the biggest difference from animals [55,56]. Previous studies have shown that TOR signaling is closely related to chloroplast development and photosynthesis in plants [33,57,58]. Photosynthetic absorption of CO_2_ increased TOR activity, which in turn the enhanced TOR activity further promoted photosynthesis in *C. reinhardtii* [58]. Most DEGs involving chloroplast development and photosynthesis, such as thylakoid, porphyrin and chlorophyll biosynthesis, and photosynthesis, were down-regulated under ApTOR inhibition by AZD8055 (Table 2), showing that ApTOR had important effects on chloroplast development and photosynthesis of *A. pyrenoidosa*.

Protein degradation is mainly mediated by the ubiquitin/26S proteasome pathway and autophagy [59,60]. In this study, we found that ApTOR inhibition activates autophagy and ubiquitin mediated proteolysis pathway in *A. pyrenoidosa* (Table 3), promoting catabolism of protein. However, genes related to ribosome synthesis were significantly down-regulated in the RNA-seq data, thus inhibiting protein synthesis. These results indicated that ApTOR is involved in regulating protein synthesis and catabolism by multiple metabolic pathways in *A. pyrenoidosa*. Furthermore, the transcriptome data showed that ApTOR controls various intracellular metabolism and signaling pathways in *A. pyrenoidosa*. Inhibition of ApTOR activity resulted in disorders of carbon and nitrogen metabolism, protein and fatty acid metabolism and TCA cycle, which further inhibited the cell growth of *A. pyrenoidosa*.

## 4. Materials and Methods

### 4.1. Algae and Growth Condition

The strain of *A. pyrenoidosa* (FACHB-9) used in this study was purchased from the Institute of Hydrobiology, Chinese Academy of Sciences (Wuhan, China). *A. pyrenoidosa* was cultured in BG11 liquid medium supplemented with 20 g.L^−1^ glucose under 28 °C, 2000 lux continuous light, and 180 rpm.

### 4.2. Treatment of Auxenochlorella Pyrenoidosa by TOR Inhibitors

The *A. pyrenoidosa* cells was inoculated into a 50 mL BG11 liquid medium supplemented with different concentrations of TOR inhibitors (rapamycin, AZD8055, KU0063794, Torin1) and incubated at 28 °C, 2000 lux continuous light, and 180 rpm. The cell density at 680 nm optical density (OD680) was measured with a Microplate Reader (Biotek EpochTM2, Winooski, VT, USA) at 0, 2, 4 and 6 days.

To test whether *A. pyrenoidosa* cells were killed by high concentrations of AZD8055. *A. pyrenoidosa* cells were treated with 1, 5 and 10 μM AZD8055 for 4 days, and AZD8055 was removed from the medium, then the pellet was resuspended with BG11 and adjusted to the same OD value. Meanwhile, the removed supernatant containing different concentrations of AZD8055 was added into fresh *A. pyrenoidosa* cells. The phenotype was observed after culturing with or without AZD8055 for 4 days.

### 4.3. Phylogenetic Tree Analysis

Homologous sequences from different species were aligned by ClustalX software. Phylogenetic tree was generated from the Neighbor-Joining method by MEGA 4 software, and Poisson correction model was used to compute genetic distance. TpTOR (XP_002293107.1), CrTOR (XP_042921379.1), PtTOR (XP_002181617.1), AtTOR (NP_175425.2), HsTOR (NP_001373429.1), ScTOR1 (NP_012600.1), ScTOR2 (NP_012719.2), CrFKBP12 (XP_001693615.1), AtFKBP12 (NP_201240.1), StFKBP12 (XP_006351741.1), OsFKBP12 (XP_015625368.1), SpFKBP12 (NP_595257.1), HsFKBP12 (NP_000792.1) and ScFKBP12 (NP_014264.1) protein sequences were download from NCBI database.

### 4.4. Construction of the RNA-seq Library and Transcriptome Sequencing

*A. pyrenoidosa* was cultured in 50 mL BG11 liquid medium supplemented with 20 g·L^−1^ glucose at 28 °C, 2000 lux continuous light, and 180 rpm for 4 days. Then, final-concentration 5 μM AZD8055 and equivalent DMSO were added into the alga solution for 24 h, and algal cells were precipitated by centrifugation and collected. Three independent biological replicates were performed for each treatment. Total RNA of *A. pyrenoidosa* treated with AZD8055 or DMSO was extracted by Plant RNA extraction kit (TIANGEN, Beijing, China). The RNA library was constructed using NEBNext^®^ Ultra TMRNA Library Prep Kit (NEB, Boston, MA, USA) by Tianjin Novogene Bioinformatics Technology Co., Ltd. Qualified library was sequenced on an Illumina Novaseq 6000 platform and 150 bp paired-end reads were generated. Clean reads were obtained by filtering the raw data.

### 4.5. Transcriptome Assembly, Annotation and Differential Expression Analysis

After obtaining clean reads, the Trinity software (V2.6.6, Marlborough, MA, USA) [61] was used to spliced clean reads to obtain reference sequences for subsequent analysis. Diamond software (V0.9.13.114, Tübingen, Germany) [62] was used to match the gene sequence into the protein database for functional annotation. Using gene function annotations information from major databases, including NR, GO, KEGG, Pfam, KOG/COG, and Swiss-prot databases, the spliced genes were annotated. DESeq2 R package (1.20.0) [63] was used to analyze the differentially expressed genes (DEGs) between AZD8055 treatment and DMSO control. *P*-adj < 0.05 and | Log2 (Fold change)|> 1 were set as the threshold values of gene differential expression. GO and KEGG plant databases were used to predict the function of genes and describe the gene products, and the annotation information related to plants was selected for GO and KEGG pathway enrichment. Goseq (V1.10.0, Parkville, Australia) and KOBAS (V2.0.12, Beijing, China) software were used for GO and KEGG pathway enrichment analysis of DEGs, respectively [64,65].

### 4.6. Quantitative Real-Time PCR (qRT-PCR) Validation

To verify reliability of transcriptome data, qRT-PCR was used to quantify the expression levels of 10 randomly selected genes. CDS sequences of the genes were derived from transcriptome sequencing data, and the corresponding specific primers were presented in Supplementary Table S5. *ApActin* (*Cluster-495.7101*) was used as a reference gene. RNA from *A. pyrenoidosa* that was processed in the same batch as transcriptome sequencing was selected for qRT-PCR. Relative expression levels of genes were assayed by two-step RT-PCR analysis using the Bio-Rad CFX96 Manager software (BIO-RAD, Hercules, CA, USA). Reaction was performed in a final volume of 20 µL containing 10 µL of 2 × SYBR Green PCR Mastermix (Solarbio, Beijing, China). The relative RNA products of the genes were analyzed using the formula 2^−^^ΔΔCT^.

## 5. Conclusions

In conclusion, this study revealed the conserved ApTOR signaling in *A. pyrenoidosa* and elucidated the effects of TOR inhibitors on the growth of *A. pyrenoidosa*. Transcriptome data results showed that ApTOR is involved in regulating chloroplast development, photosynthesis and intracellular metabolism in *A. pyrenoidosa*, and ApTOR promotes the cell growth of *A. pyrenoidosa* by regulating various signaling pathways and intracellular metabolic processes. This study provides some insights into the function of ApTOR in *A. pyrenoidosa*.

## Figures and Tables

**Table 1:** The putative components of TOR signaling pathway in *Auxenochlorella pyrenoidosa*.

Protein Name	*Chlamydomonas reinhardtii*	*Auxenochlorella pyrenoidosa*	Identity (%)
Target of rapamycin (TOR)	CrTOR	ApTOR like	58
Regulatory associate protein of TOR (RAPTOR)	CrRAPTOR	ApRAPTOR like	37
Lethal with SEC-13 protein 8 (LST8)	CrLST8	ApLST8 like	81
FK506-binding protein 12 kDa (FKBP12)	CrFKBP12	ApFKBP12 like	43
Ribosomal protein S6 kinase (S6K)	CrS6K	ApS6K like	45
Ribosome protein small subunit 6 (RPS6)	CrRPS6	ApRPS6 like	78
Transcription factor E2F alpha (E2FA)	CrE2FA	ApE2FA like	39
Translation initiation factor 2 alpha subunit (eIF2α)	CreIF2α	ApeIF2α like	72
Type 2A phosphatase associated protein 46 (TAP46)	CrTAP46	ApTAP46 like	40
Autophagy protein 1 (ATG1)	CrATG1	ApATG1 like	39
Autophagy protein 13 (ATG13)	CrATG13	ApATG13 like	32

**Table 2:** Differentially expressed genes in the photosynthetic process.

Gene ID	Log_2_ (Fold Change)	P-adj	KO Name	KO Description
**Photosynthesis**				
Cluster-495.6756	−5.9733	5.56 × 10^−90^	PETE	Plastocyanin
Cluster-498.0	−5.6390	3.47 × 10^−5^	PSBE	Photosystem II cytochrome b559 subunit α
Cluster-495.7678	−5.6230	6.53 × 10^−124^	PSAN	Photosystem I subunit psan
Cluster-495.7002	−5.4544	9.90 × 10^−104^	PSAH	Photosystem I subunit VI
Cluster-495.6324	−5.3253	2.42 × 10^−70^	PSBP	Photosystem II oxygen-evolving enhancer 2
Cluster-495.8198	−5.2049	9.45 × 10^−3^	ATPF0B	F-type H+ transporting ATPase subunit b
Cluster-495.827	−4.6442	6.89 × 10^−4^	PETA	Apocytochrome f
Cluster-495.10228	−4.6211	4.34 × 10^−2^	PSBJ	Photosystem II psbj protein
Cluster-495.5726	−4.0208	1.05 × 10^−31^	PSBS	Photosystem II 22kda protein
Cluster-495.5249	−4.0063	1.02 × 10^−46^	PSB27	Photosystem II Psb27 protein
Cluster-495.4105	−3.9952	2.49 × 10^−2^	ATPF1A	F-type H+-transporting atpase subunit alpha
Cluster-495.6958	−3.8390	5.22 × 10^−43^	PSAF	Photosystem I subunit III
Cluster-495.7332	−3.7857	2.68 × 10^−3^	PSBC	Photosystem II CP43 chlorophyll apoprotein
Cluster-495.6505	−3.4964	1.36 × 10^−28^	PSAK	Photosystem I subunit X
Cluster-495.6979	−3.3834	1.87 × 10^−23^	PSBR	Photosystem II 10kda protein
Cluster-495.8002	−3.2884	4.79 × 10^−6^	ATPF1B	F-type H+-transporting atpase subunit beta
Cluster-495.6124	−3.1305	3.85 × 10^−3^	PETB	Cytochrome b6
Cluster-495.7894	−3.0592	3.40 × 10^−17^	PSAB	Photosystem I P700 chlorophyll a apoprotein
Cluster-495.1037	−2.9414	3.35 × 10^−20^	PSBP	Photosystem II oxygen-evolving enhancer 2
Cluster-495.4143	−2.8547	8.51 × 10^−45^	PSAD	Photosystem I subunit II
Cluster-495.6035	−2.8402	3.44 × 10^−36^	PSAL	Photosystem I subunit XI
Cluster-495.4194	−2.7017	7.15 × 10^−18^	PSAO	Photosystem I subunit psao
Cluster-495.4418	−2.3110	2.12 × 10^−21^	PSAG	Photosystem I subunit V
Cluster-495.5609	−2.2904	4.52 × 10^−18^	PSBO	Photosystem II oxygen-evolving enhancer 1
Cluster-495.4835	−1.7041	5.65 × 10^−12^	PETJ	Cytochrome c6
Cluster-495.6263	−1.5457	9.25 × 10^−13^	PETH	Ferredoxin--NADP+ reductase
Cluster-495.7190	−1.4895	5.90 × 10^−11^	PSBY	Photosystem II psby protein
Cluster-495.4933	−1.2359	4.99 × 10^−8^	PSB28	Photosystem II 13kda protein
Cluster-495.7871	1.1214	1.83 × 10^−5^	PETF	Ferredoxin
**Photosynthesis-antenna proteins**				
Cluster-495.6691	−7.0421	1.03 × 10^−108^	LHCA4	Photosystem I chlorophyll a/b binding protein 4
Cluster-495.3640	−5.9832	4.43 × 10^−122^	LHCA3	Photosystem I chlorophyll a/b binding protein 3
Cluster-495.6564	−5.2717	1.81 × 10^−78^	LHCA1	Photosystem I chlorophyll a/b binding protein 1
Cluster-495.5144	−4.5671	6.62 × 10^−78^	LHCB4	Photosystem II chlorophyll a/b binding protein 4
Cluster-495.6386	−4.1987	1.03 × 10^−51^	LHCB1	Photosystem II chlorophyll a/b binding protein 1
Cluster-495.5553	−3.9840	1.96 × 10^−76^	LHCA4	Photosystem I chlorophyll a/b binding protein 4
Cluster-495.6485	−3.9329	1.45 × 10^−40^	LHCB5	Photosystem II chlorophyll a/b binding protein 5
Cluster-495.6349	−3.4326	3.63 × 10^−33^	LHCB1	Photosystem II chlorophyll a/b binding protein 1
Cluster-495.8808	−2.5721	2.26 × 10^−2^	LHCB2	Photosystem II chlorophyll a/b binding protein 2
**Carbon fixation in photosynthetic organisms**				
Cluster-495.5099	−5.0936	5.78 × 10^−121^	PGK	Phosphoglycerate kinase
Cluster-495.2276	−4.3041	1.39 × 10^−43^	ALDO	Fructose-bisphosphate aldolase, class I
Cluster-495.7895	−4.0597	1.13 × 10^−9^	GOT2	Aspartate aminotransferase
Cluster-495.2903	−3.5833	1.72 × 10^−36^	MDH2	Malate dehydrogenase
Cluster-495.5815	−3.0446	6.82 × 10^−42^	PRKB	Phosphoribulokinase
Cluster-495.5677	−2.9728	3.99 × 10^−29^	PPDK	Pyruvate, orthophosphate dikinase
Cluster-495.5005	−2.8583	3.63 × 10^−42^	TPI	Triosephosphate isomerase (TIM)
Cluster-495.5217	−2.7963	1.37 × 10^−29^	ALDO	Fructose-bisphosphate aldolase, class I
Cluster-495.4546	−2.7251	3.02 × 10^−38^	TPI	Triosephosphate isomerase (TIM)
Cluster-495.5985	−2.5229	6.25 × 10^−42^	MDH1	Malate dehydrogenase
Cluster-495.3332	−2.5073	2.29 × 10^−19^	RPIA	Ribose 5-phosphate isomerase A
Cluster-495.1259	−2.5056	8.33 × 10^−6^	PCKA	Phosphoenolpyruvate carboxykinase
Cluster-495.4145	−1.8990	6.39 × 10^−16^	MDH2	Malate dehydrogenase
Cluster-495.4967	−1.7549	3.89 × 10^−11^	MAEB	Malate dehydrogenase (NADP+)
Cluster-495.3492	−1.6252	7.78 × 10^−7^	MAEB	Malate dehydrogenase (NADP+)
Cluster-495.5038	−1.6083	3.40 × 10^−17^	TKTA	Transketolase
Cluster-495.6521	−1.6040	1.68 × 10^−14^	GAPDH	Glyceraldehyde-3-phosphate dehydrogenase
Cluster-495.6227	−1.2768	9.08 × 10^−9^	GAPDH	Glyceraldehyde 3-phosphate dehydrogenase
Cluster-495.3372	−1.1984	1.25 × 10^−4^	PPC	Phosphoenolpyruvate carboxylase
Cluster-495.5601	−1.0961	2.63 × 10^−9^	E3.1.3.37	Sedoheptulose-bisphosphatase
Cluster-495.2869	−1.0914	3.29 × 10^−7^	FBP	Fructose-1,6-bisphosphatase I
Cluster-495.6190	−1.0435	5.51 × 10^−5^	GAPDH	Glyceraldehyde 3-phosphate dehydrogenase
Cluster-495.5207	1.1296	7.49 × 10^−7^	E1.1.1.39	Malate dehydrogenase (decarboxylating)
Cluster-495.7215	1.4168	6.87 × 10^−8^	GGAT	Glutamate-glyoxylate aminotransferase

**Table 3:** Differentially expressed genes in protein catabolism.

Gene ID	Log_2_ (Fold Change)	P-adj	KO Name	KO Description
**Regulation of autophagy**				
Cluster-495.165	1.6278	3.68 × 10^−2^	SnRK1α	SNF1-related protein kinase 1 α subunit
Cluster-495.6489	1.5520	9.00 × 10^−13^	ATG7	Autophagy-related protein 7
Cluster-495.4583	1.3266	5.06 × 10^−5^	ATG3	Autophagy-related protein 3
Cluster-495.7507	1.2287	1.39 × 10^−6^	ATG16L1	Autophagy-related protein 16-1
Cluster-495.6822	1.1679	3.45 × 10^−6^	SnRK1α	SNF1-related protein kinase 1 α subunit
Cluster-495.7877	1.1533	1.91 × 10^−7^	ATG101	Autophagy-related protein 101
Cluster-495.4705	1.0000	4.53 × 10^−6^	ATG11	Autophagy-related protein 11
Cluster-495.2673	−1.6051	8.07 × 10^−6^	ATG12	Autophagy-related protein 12
**Ubiquitin mediated proteolysis**				
Cluster-495.5469	2.9686	4.17 × 10^−21^	UBLE1B	Ubiquitin-like 1-activating enzyme E1 B
Cluster-495.3804	2.7399	2.91 × 10^−53^	UBE2A	Ubiquitin-conjugating enzyme E2 A
Cluster-495.4516	2.3622	8.08 × 10^−26^	ERCC8	DNA excision repair protein ERCC8
Cluster-495.4258	1.7941	1.47 × 10^−13^	CDH1	Cell division cycle 20-like protein 1
Cluster-495.3283	1.7552	2.29 × 10^−19^	CUL3	Cullin 3
Cluster-495.5596	1.7515	2.80 × 10^−2^	UBLE1B	Ubiquitin-like 1-activating enzyme E1 B
Cluster-495.8038	1.4688	1.57 × 10^−16^	CUL1	Cullin 1
Cluster-495.4275	1.4562	6.98 × 10^−11^	SKP1	S-phase kinase-associated protein 1
Cluster-495.6597	1.4529	1.99 × 10^−6^	UBE2E	Ubiquitin-conjugating enzyme E2 E
Cluster-495.4573	1.2755	1.57 × 10^−9^	UBE1	Ubiquitin-activating enzyme E1
Cluster-495.4611	1.2633	8.88 × 10^−9^	CUL4	Cullin 4
Cluster-495.2951	1.1905	1.28 × 10^−8^	UBE1C	Ubiquitin-activating enzyme E1 C
Cluster-495.8466	1.1285	6.54 × 10^−4^	RBX1	RING-box protein 1
Cluster-495.1824	1.0291	3.12 × 10^−5^	PPIL2	Peptidyl-prolyl cis-trans isomerase-like 2
Cluster-495.4193	1.0217	1.66 × 10^−3^	RBX1	RING-box protein 1
Cluster-495.1695	−1.1161	1.39 × 10^−3^	FANCL	E3 ubiquitin-protein ligase FANCL
Cluster-495.1057	−1.2830	1.16 × 10^−4^	UBE3A	Ubiquitin-protein ligase E3 A
Cluster-495.7130	−1.6390	1.07 × 10^−10^	UBLE1A	Ubiquitin-like 1-activating enzyme E1 A
Cluster-495.9644	−1.7193	5.31 × 10^−3^	UBE2S	Ubiquitin-conjugating enzyme E2 S

**Table 4:** Differentially expressed genes in the TCA cycle.

Gene ID	Log2 (Fold Change)	P-adj	KO Name	KO Description
Cluster-495.2903	−3.5833	1.72 × 10^−36^	MDH2	Malate dehydrogenase
Cluster-495.5985	−2.5229	6.25 × 10^−42^	MDH1	Malate dehydrogenase
Cluster-495.1259	−2.5056	8.33 × 10^−6^	PCKA	Phosphoenolpyruvate carboxykinase
Cluster-495.4145	−1.8990	6.39 × 10^−16^	MDH2	Malate dehydrogenase
Cluster-495.5734	−1.6082	5.41 × 10^−16^	LSC1	Succinyl-CoA synthetase alpha subunit
Cluster-495.5974	−1.5814	3.82 × 10^−15^	ACO	Aconitate hydratase
Cluster-495.4348	−1.4680	3.68 × 10^−8^	PDHD	Dihydrolipoamide dehydrogenase
Cluster-495.4853	−1.2993	1.45 × 10^−10^	DLST	α-oxoglutarate dehydrogenase E2
Cluster-495.6949	−1.2720	1.91 × 10^−5^	PDHC	Pyruvate dehydrogenase E2
Cluster-495.6523	−1.2587	7.65 × 10^−7^	FUMC	Fumarate hydratase, class II
Cluster-495.5492	−1.2187	2.33 × 10^−8^	IDH1	Isocitrate dehydrogenase
Cluster-495.6105	−1.2067	8.64 × 10^−11^	LSC2	Succinyl-CoA synthetase beta subunit
Cluster-495.2778	−1.1371	2.54 × 10^−6^	SDHD	Succinate dehydrogenase subunit
Cluster-495.5250	−1.1254	1.39 × 10^−6^	OGDH	α-oxoglutarate dehydrogenase E1

## Data Availability

The transcriptome data have been deposited in the NCBI Sequence Read Archive under accession number PRJNA841794.

## Supplementary Materials

The following supporting information can be downloaded at: https://www.mdpi.com/article/10.3390/ijms231911309/s1.
