# Supplementary material for: Target of Rapamycin Regulates Photosynthesis and Cell Growth in Auxenochlorella pyrenoidosa
Source: Int J Mol Sci. 2022 Sep 25;23(19):11309. doi: 10.3390/ijms231911309 (PMC9569773; doi:10.3390/ijms231911309)
Supplement: Supplementary file 1 [file ijms-23-11309-s001.zip › ijms-1872255-supplementary.pdf]

Target of rapamycin regulates photosynthesis and cell growth in *Auxenochlorella pyrenoidosa*

Tingting Zhu[1,2,†], Linxuan Li[1,2,3,†], Huimin Chang[3], Jiasui Zhan[4,*] and Maozhi Ren[1,2,3,*]

Supplementary Figures and Tables

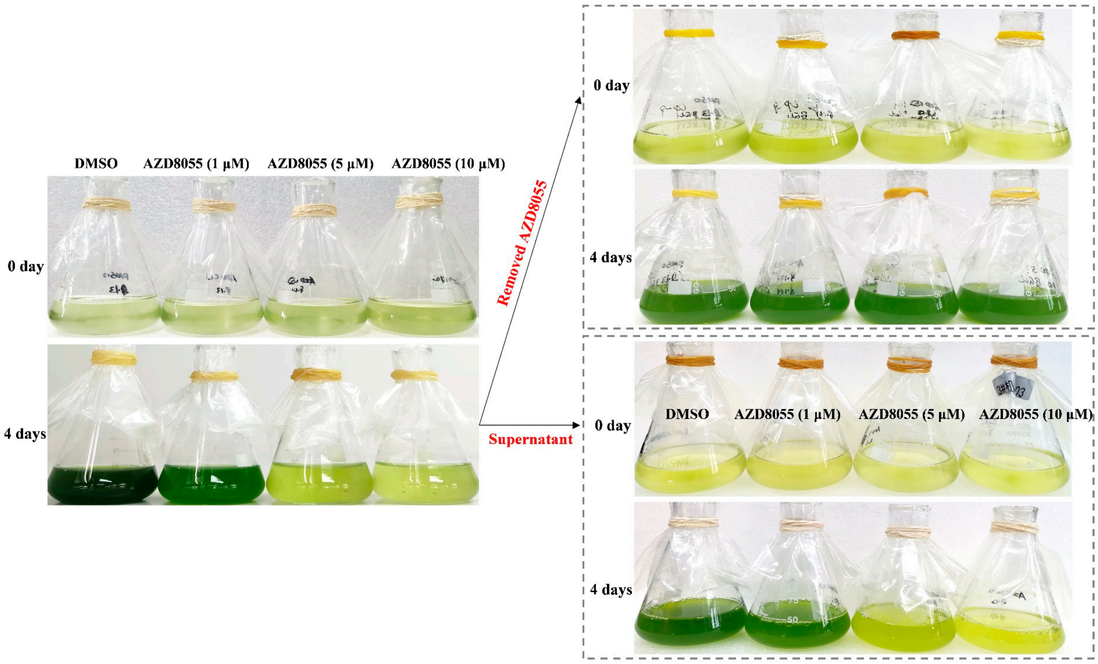

Supplementary Figure S1. Inhibition of ApTOR kinase activity by AZD8055 prevents cell division in *Auxenochlorella pyrenoidosa*. *A. pyrenoidosa* cells were treated with 1, 5 and 10 μM AZD8055 for 4 days, and AZD8055 was removed from the medium, then the pellet was resuspended with BG11 and adjusted to the same OD value. Meanwhile, the removed supernatant containing different concentrations of AZD8055 was added into fresh *A. pyrenoidosa* cells. The phenotype was observed after culturing with or without AZD8055 for 4 days.

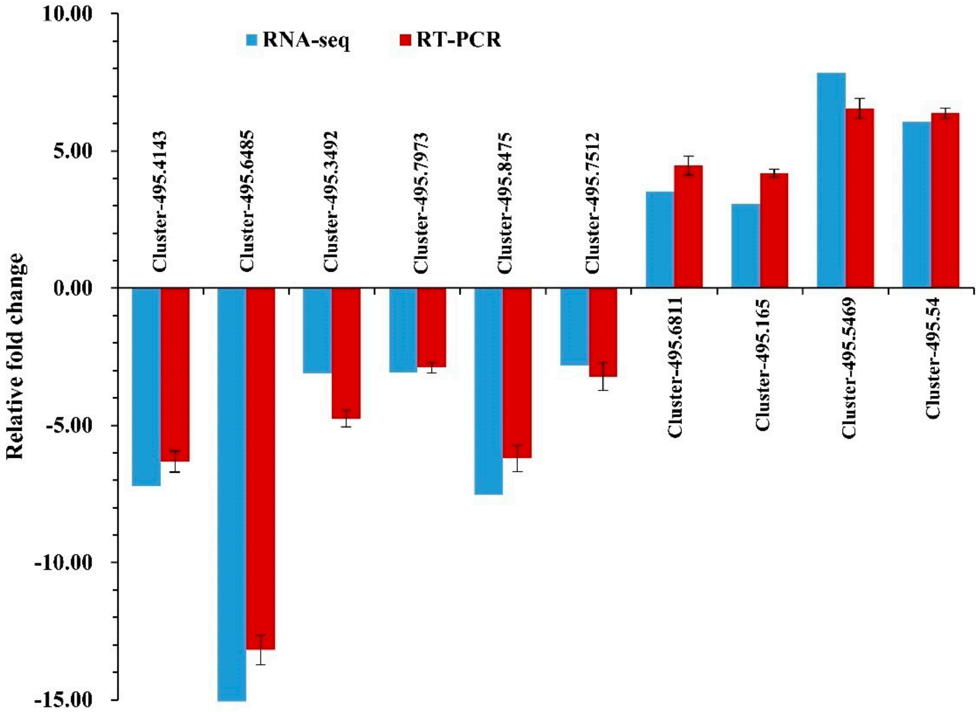

Supplementary Figure S2. Gene expression levels of differentially expressed genes both in the real time PCR and RNA-seq data. The data were expressed as the mean ± SD of three independent experiments.

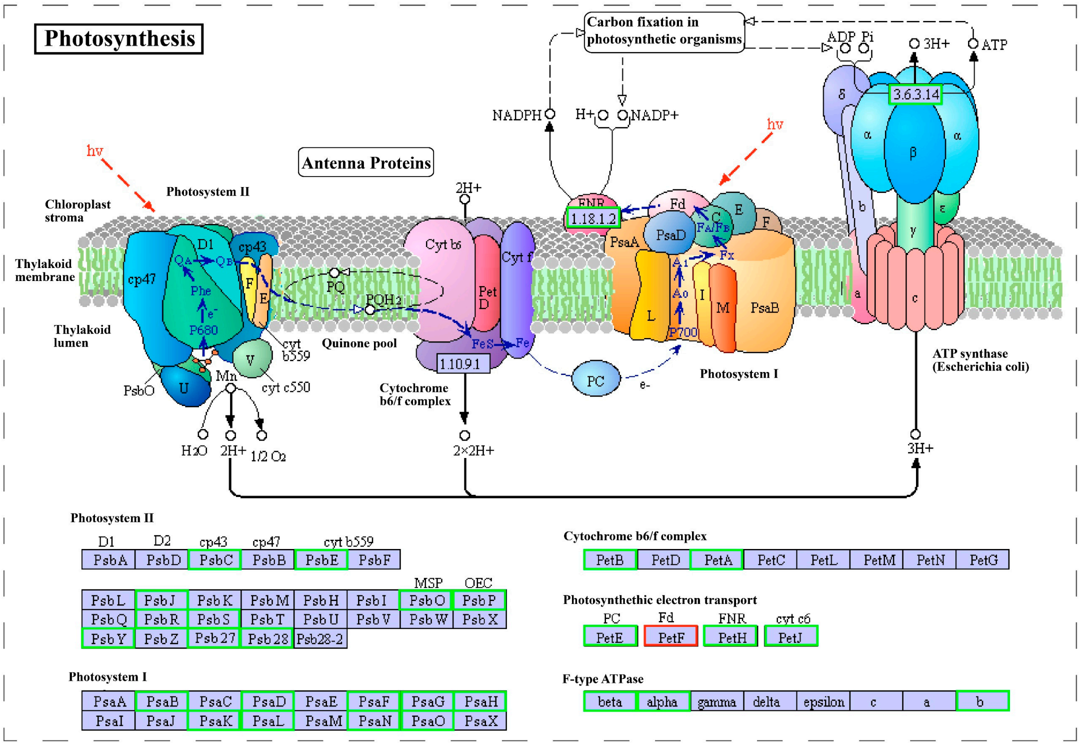

Supplementary Figure S3. ApTOR regulates photosynthesis in *Auxenochlorella pyrenoidosa*. Green boxes denote down-regulated genes, and red boxes represent up-regulated genes.

Supplementary Table S1. Summary of the RNA-seq data

Sample	Raw Reads	Clean Reads	Clean Bases	Error Rate (%)	Q20 (%)	Q30 (%)	GC Content (%)
DMSO_1	25408251	23944899	7.18 G	0.03	96.72	92.14	66.72
DMSO_2	23103782	21669919	6.50 G	0.03	96.87	92.53	66.61
DMSO_3	23996010	22234144	6.67 G	0.03	96.91	92.50	66.54
AZD_1	27706690	26271415	7.88 G	0.03	97.16	92.96	66.23
AZD_2	23889753	23363899	7.01 G	0.03	96.65	92.00	66.45
AZD_3	23169941	22681356	6.80 G	0.03	97.60	94.12	66.45

Supplementary Table S2. DEGs in chloroplast biogenesis and development

Gene ID	Log_2 (Fold change)	P-adj	KO Name	KO Description
**Porphyrin and chlorophyll biosynthesis**				
Cluster-495.6688	-4.7867	7.71E-73	chlP, bchP	Geranylgeranyl diphosphate reductase
Cluster-495.7988	-4.3298	1.63E-18	bchM, chlM	Geranylgeranyl reductase
Cluster-495.3211	-3.8931	1.64E-42	hemL	Glutamate semialdehyde aminomutase
Cluster-495.6256	-3.2222	1.75E-37	chlH, bchH	Magnesium chelatase subunit H
Cluster-495.7219	-3.2049	6.40E-17	por	Protochlorophyllide reductase
Cluster-495.678	-3.1809	5.39E-03	UROS	Uroporphyrinogen-III synthase
Cluster-495.9572	-3.0728	6.34E-11	CPOX	Coproporphyrinogen III oxidase
Cluster-495.8937	-2.9705	1.72E-20	HMBS	Hydroxymethylbilane synthase
Cluster-495.6372	-2.6989	3.57E-18	hemB	Porphobilinogen synthase
Cluster-495.7885	-2.0294	9.90E-12	chlD, bchD	Magnesium chelatase subunit D
Cluster-495.3171	-2.0066	5.03E-13	CPOX	Coproporphyrinogen III oxidase
Cluster-495.7024	-1.9459	1.91E-11	UROD	Uroporphyrinogen decarboxylase
Cluster-495.6966	-1.8103	1.84E-17	acsF, chlE	Magnesium-protoporphyrin cyclase
Cluster-495.7973	-1.6219	6.59E-06	CAO	Chlorophyllide a oxygenase
Cluster-495.5687	-1.1905	1.14E-10	hemA	Glutamyl-tRNA reductase
Cluster-495.4976	-1.1750	4.45E-05	CPOX	Coproporphyrinogen III oxidase
**Thylakoid**				
Cluster-495.8121	-6.8519	7.01E-07	--	--

Cluster-495.851	-6.5033	1.03E-06	Ycf12	--
Cluster-495.9278	-5.7186	2.69E-04	--	CONSTANS-like B-box zinc finger
Cluster-498.0	-5.6390	3.47E-05	psbE	Photosystem II cytochrome b559
Cluster-495.7678	-5.6230	6.53E-124	psaN	Photosystem I subunit PsaN
Cluster-495.7002	-5.4544	9.90E-104	psaH	Photosystem I subunit VI
Cluster-495.6324	-5.3253	2.42E-70	psbP	Oxygen-evolving enhancer protein 2
Cluster-495.8252	-5.2531	1.49E-29	--	Photosystem II
Cluster-495.9999	-4.6992	8.06E-05	--	Zinc- peroxisomal
Cluster-495.827	-4.6442	6.89E-04	petA	Apocytochrome f
Cluster-495.10228	-4.6211	4.34E-02	psbJ	Photosystem II PsbJ protein
Cluster-753.0	-4.4128	2.35E-03	--	Photosystem II repair PSB27
Cluster-495.866	-4.0511	1.28E-08	rpsG	Small subunit protein S7
Cluster-495.5249	-4.0063	1.02E-46	psb27	Photosystem II Psb27 protein
Cluster-495.1246	-3.9817	6.20E-11	--	Photosystem I reaction center subunit I
Cluster-495.6056	-3.8938	8.32E-43	--	Chloroplast RF12
Cluster-495.6958	-3.8390	5.22E-43	psaF	Photosystem I subunit III
Cluster-495.7332	-3.7857	2.68E-03	psbC	Photosystem II chlorophyll apoprotein
Cluster-495.10100	-3.6324	7.09E-03	--	Photosystem II repair PSB27
Cluster-495.6995	-3.5387	1.57E-17	--	UPF0603 chloroplastic
Cluster-495.6505	-3.4964	1.36E-28	psaK	Photosystem I subunit X
Cluster-495.2603	-3.4907	3.76E-24	--	--
Cluster-495.6979	-3.3834	1.87E-23	psbR	Photosystem II 10kDa protein
Cluster-495.8520	-3.3777	6.57E-03	--	--
Cluster-495.9180	-3.1148	2.64E-16	rplL	Large subunit protein L7
Cluster-495.7894	-3.0592	3.40E-17	psaB	Photosystem I chlorophyll a apoprotein
Cluster-495.1037	-2.9414	3.35E-20	psbP	Oxygen-evolving enhancer protein 2
Cluster-495.4143	-2.8547	8.51E-45	psaD	Photosystem I subunit II
Cluster-495.6035	-2.8402	3.44E-36	psaL	Photosystem I subunit XI
Cluster-495.6788	-2.7720	1.49E-28	EIF2S1	Translation initiation factor 2 subunit 1
Cluster-495.2701	-2.4827	1.02E-15	--	--
Cluster-495.4418	-2.3110	2.12E-21	psaG	Photosystem I subunit V
Cluster-495.5609	-2.2904	4.52E-18	psbO	Oxygen-evolving enhancer protein 1
Cluster-495.9560	-2.2550	6.99E-03	--	Photosystem II cytochrome b559 alpha

Cluster-495.8596	-1.9710	5.46E-04	--	PsbP chloroplastic
Cluster-495.9554	-1.9043	3.59E-03	--	Photosystem II reaction center psb28
Cluster-495.6767	-1.8672	1.71E-05	--	--
Cluster-495.5836	-1.8644	3.62E-12	--	Slug 5-like
Cluster-495.4536	-1.8049	2.00E-16	--	IMPACT family member
Cluster-495.3452	-1.7610	2.50E-09	clpB	Clp protease ATP-binding subunit
Cluster-495.7190	-1.4895	5.90E-11	psbY	Photosystem II PsbY protein
Cluster-495.3743	-1.4058	6.61E-12	--	--
Cluster-495.6413	-1.3246	6.47E-08	--	--
Cluster-495.4933	-1.2359	4.99E-08	psb28	Photosystem II 13kDa protein
Cluster-495.1459	1.0994	4.45E-04	COL3A	Collagen, type III, alpha
Cluster-495.6239	1.1139	2.46E-05	--	Photosystem I reaction center subunit
Cluster-495.3769	1.1750	1.33E-08	--	reductase SDR family member 7
Cluster-495.4010	1.2834	7.21E-08	PDIA6,	Potein disulfide-isomerase A6
Cluster-495.5479	1.5785	2.07E-12	--	Photosystem I reaction center subunit
Cluster-495.3654	1.6019	4.22E-19	--	Dihydrolipoyl dehydrogenase

Supplementary Table S3. DEGs in "Ribosome biogenesis" pathway

Gene ID	Log_2 (Fold change)	P-adj	KO Name	KO Description
**Ribosome biogenesis**				
Cluster-829.0	-4.7112	3.61E-02	RPL2	50S ribosomal protein L2
Cluster-495.244	-4.1285	3.49E-02	RPL34	60S ribosomal protein L34
Cluster-495.866	-4.0511	1.28E-08	RPS7	Small subunit ribosomal protein S7
Cluster-495.7112	-3.9511	8.68E-22	CTR1	Serine threonine- kinase CTR1
Cluster-495.1410	-3.7252	4.04E-17	RPS6	Small subunit ribosomal protein S6
Cluster-495.7354	-3.4826	1.04E-29	--	Hypothetical protein
Cluster-495.6652	-3.2037	3.59E-22	EIF3	Translation initiation factor IF-3
Cluster-495.9180	-3.1148	2.64E-16	RPL7	Large subunit ribosomal protein L7
Cluster-495.8475	-2.9179	1.83E-10	RPL18A	Large subunit ribosomal protein L18A
Cluster-495.6595	-2.5200	5.02E-35	--	Hypothetical protein
Cluster-495.1096	-2.1232	2.52E-03	--	--
Cluster-495.6333	-2.0865	5.66E-10	cysC	Adenylylsulfate kinase
Cluster-495.1341	-2.0406	3.06E-08	yqfG	Probable rrna maturation factor
Cluster-495.10141	-1.8808	2.06E-05	POLH	DNA polymerase
Cluster-495.7376	-1.8124	5.85E-08	RPL19	Large subunit ribosomal protein L19
Cluster-495.702	-1.5495	7.78E-03	RPL4	Large subunit ribosomal protein L4
Cluster-495.9613	-1.5297	2.12E-03	RPSSD--	Ribosomal large subunit synthase D

Cluster-495.3907	-1.4939	3.84E-15	--	Hypothetical protein
Cluster-495.1789	-1.4250	1.28E-05	SNRPE	Small nuclear ribonucleoprotein E
Cluster-495.6690	-1.3820	1.77E-07	--	--
Cluster-495.2121	-1.3778	3.47E-02	RPL3	Large subunit ribosomal protein L3
Cluster-495.5110	-1.3548	8.14E-09	INO1	Myo-inositol-1-phosphate synthase
Cluster-495.6827	-1.1888	3.36E-08	RPS15	Small subunit ribosomal protein S15
Cluster-495.2500	-1.1802	7.87E-05	--	Hypothetical protein
Cluster-495.7267	-1.1693	3.64E-04	--	--
Cluster-495.3701	-1.0849	5.30E-05	UTP21	U3 small nucleolar RNA-associated protein 21
Cluster-495.5751	1.0353	5.02E-03	PL34	Large subunit ribosomal protein l34e
Cluster-495.4882	1.0366	6.31E-03	PL14	Large subunit ribosomal protein l14e
Cluster-495.5996	1.0490	4.05E-03	PS29	Small subunit ribosomal protein s29e
Cluster-495.8869	1.0601	3.27E-02	--	--
Cluster-495.3195	1.0677	4.18E-07	RPS12	Ribosomal protein S12
Cluster-495.5637	1.0915	9.34E-03	RPS6	Small subunit ribosomal protein s6e
Cluster-495.5611	1.0976	6.64E-04	PL13A	Large subunit ribosomal protein L13A
Cluster-495.5652	1.1674	7.95E-04	RPL31	Large subunit ribosomal protein l31e
Cluster-495.1559	1.1713	1.64E-04	--	--
Cluster-495.8036	1.2193	3.30E-03	--	Ribosome-binding factor A
Cluster-495.6197	1.2338	5.38E-05	RPL22	Large subunit ribosomal protein l22e
Cluster-495.3935	1.3084	5.31E-12	THOC2	THO complex subunit 2
Cluster-495.6944	1.3088	6.91E-10	UTP10	U3 small nucleolar RNA-associated protein 10
Cluster-495.5550	1.3294	1.97E-07	RPS23	Small subunit ribosomal protein s23e

Supplementary Table S4. DEGs in carbon, amino acid and fatty acid metabolism

Gene ID	Log_2 (Fold change)	P-adj	KO Name	KO Description
**Carbon metabolism**				
Cluster-495.5099	-5.0936	5.78E-121	PGK, pgk	Phosphoglycerate kinase
Cluster-495.2276	-4.3041	1.39E-43	ALDO	Fructose-bisphosphate aldolase
Cluster-495.7895	-4.0597	1.13E-09	GOT2	Aspartate aminotransferase
Cluster-495.4325	-3.8674	1.08E-66	gapN	Glyceraldehyde-3-phosphate dehydrogenase (NADP+)
Cluster-495.2903	-3.5833	1.72E-36	MDH2	Malate dehydrogenase
Cluster-495.6407	-3.5684	6.23E-08	cysE	Serine O-acetyltransferase
Cluster-495.5815	-3.0446	6.82E-42	PRK, prkB	Phosphoribulokinase

Cluster-495.5677	-2.9728	3.99E-29	ppdK	Pyruvate, orthophosphate dikinase
Cluster-495.5005	-2.8583	3.63E-42	TPI, tpiA	Triosephosphate isomerase (TIM)
Cluster-495.5217	-2.7963	1.37E-29	ALDO	Fructose-bisphosphate aldolase, class I
Cluster-495.6030	-2.7646	4.01E-22	serA, PHGDH	D-3-phosphoglycerate dehydrogenase
Cluster-495.3127	-2.7627	5.82E-26	G6PD, zwf	Glucose-6-phosphate 1-dehydrogenase
Cluster-495.4546	-2.7251	3.02E-38	TPI, tpiA	Triosephosphate isomerase (TIM)
Cluster-495.9524	-2.6593	5.00E-05	PCCA, pccA	Propionyl-coa carboxylase alpha chain
Cluster-495.5985	-2.5229	6.25E-42	MDH1	Malate dehydrogenase
Cluster-495.3332	-2.5073	2.29E-19	rpiA	Ribose 5-phosphate isomerase A
Cluster-495.1259	-2.5056	8.33E-06	pckA	Phosphoenolpyruvate carboxykinase
Cluster-495.5297	-2.4966	9.22E-21	PGD, gnd, gntZ	6-phosphogluconate dehydrogenase
Cluster-495.2017	-2.4122	1.06E-03	pfkA, PFK	6-phosphofructokinase 1
Cluster-495.5796	-2.2895	4.74E-20	accC	Acetyl-coa carboxylase subunit
Cluster-495.4722	-2.0943	3.43E-13	cysK	Cysteine synthase A
Cluster-495.6013	-1.9431	7.28E-17	cysK	Cysteine synthase A
Cluster-495.4145	-1.8990	6.39E-16	MDH2	Malate dehydrogenase
Cluster-495.4967	-1.7549	3.89E-11	maeB	Malate dehydrogenase (oxaloacetate-decarboxylating)(NADP+)
Cluster-495.2466	-1.6897	5.48E-09	RGN	Gluconolactonase
Cluster-495.3492	-1.6252	7.78E-07	E1.1.1.40, maeB	Malate dehydrogenase (oxaloacetate-decarboxylating)(NADP+)
Cluster-495.5038	-1.6083	3.40E-17	tktA, tktB	Transketolase
Cluster-495.5734	-1.6082	5.41E-16	LSC1	Succinyl-coa synthetase alpha subunit

Cluster-495.6521	-1.6040	1.68E-14	GAPA	Glyceraldehyde-3-phosphate dehydrogenase (NADP+)
Cluster-495.5974	-1.5814	3.82E-15	ACO, acnA	Aconitate hydratase
Cluster-495.3018	-1.5549	4.45E-05	PK, pyk	Pyruvate kinase
Cluster-495.7997	-1.5260	2.68E-06	PGP, PGLP	Phosphoglycolate phosphatase
Cluster-495.1844	-1.5110	3.40E-06	metF, MTHFR	Methylenetetrahydrofolate reductase
Cluster-495.7512	-1.4877	1.89E-04	talA, talB	Transaldolase
Cluster-495.4348	-1.4680	3.68E-08	DLD	Dihydrolipoamide dehydrogenase
Cluster-495.4366	-1.4535	7.04E-09	gpmI	2,3-bisphosphoglycerate-independent phosphoglycerate mutase
Cluster-495.8285	-1.4321	2.60E-02	gpmB	Probable phosphoglycerate mutase
Cluster-495.9540	-1.4116	2.97E-02	PGP, PGLP	Phosphoglycolate phosphatase
Cluster-495.6131	-1.3492	6.29E-08	GPI, pgi	Glucose-6-phosphate isomerase
Cluster-495.7334	-1.3098	1.75E-06	ADH5, adhC	Alcohol dehydrogenase
Cluster-495.4853	-1.2993	1.45E-10	DLST, sucB	2-oxoglutarate dehydrogenase E2
Cluster-495.6227	-1.2768	9.08E-09	GAPDH, gapA	Glyceraldehyde 3-phosphate dehydrogenase
Cluster-495.6949	-1.2720	1.91E-05	pdhC	Pyruvate dehydrogenase E2
Cluster-495.6523	-1.2587	7.65E-07	fumC	Fumarate hydratase, class II
Cluster-495.5492	-1.2187	2.33E-08	IDH1, IDH2	Isocitrate dehydrogenase
Cluster-495.6105	-1.2067	8.64E-11	LSC2	Succinyl-coa synthetase beta subunit
Cluster-495.3372	-1.1984	1.25E-04	ppc	Phosphoenolpyruvate carboxylase
Cluster-495.4742	-1.1802	4.04E-11	cysE	Serine O-acetyltransferase
Cluster-495.2778	-1.1371	2.54E-06	SDHD, SDH4	Succinate dehydrogenase
Cluster-495.5592	-1.1265	5.10E-05	PK, pyk	Pyruvate kinase

Cluster-495.5250	-1.1254	1.39E-06	OGDH, sucA	2-oxoglutarate dehydrogenase E1
Cluster-495.5601	-1.0961	2.63E-09	E3.1.3.37	Sedoheptulose-bisphosphatase
Cluster-495.2869	-1.0914	3.29E-07	FBP, fbp	Fructose-1,6-bisphosphatase I
Cluster-495.6190	-1.0435	5.51E-05	GAPDH	Glyceraldehyde 3-phosphate dehydrogenase
Cluster-495.9209	-1.0261	1.47E-02	ESD, fghA	S-formylglutathione hydrolase
Cluster-495.4132	-1.0164	1.28E-05	G6PD	Glucose-6-phosphate 1-dehydrogenase
Cluster-495.2709	1.0051	2.02E-03	AGXT	Alanine-glyoxylate transaminase
Cluster-495.5486	1.1196	4.71E-08	GLUD1_2	Glutamate dehydrogenase (NAD(P)+)
Cluster-495.5207	1.1296	7.49E-07	E1.1.1.39	Malate dehydrogenase
Cluster-495.5911	1.1565	7.75E-08	FDH	Formate dehydrogenase
Cluster-495.7215	1.4168	6.87E-08	GGAT	Glutamate--glyoxylate aminotransferase
Cluster-495.6647	1.4272	7.75E-08	DAK, TKFC	Triose/dihydroxyacetone kinase
Cluster-495.2622	1.7594	1.85E-22	mmsA, iolA, ALDH6A1	Malonate-semialdehyde dehydrogenase (acetylating)
Cluster-495.6811	1.8216	2.28E-12	pdhC	Pyruvate dehydrogenase E2
Cluster-495.10361	4.8009	7.22E-11	DAK, TKFC	Triose/dihydroxyacetone kinase
**Biosynthesis of amino acids**				
Cluster-870.0	-5.6022	1.42E-03	argG, ASS1	Argininosuccinate synthase
Cluster-495.7530	-5.1509	3.04E-11	TPI, tpiA	Triosephosphate isomerase (TIM)
Cluster-495.5099	-5.0936	5.78E-121	thrB1	Homoserine kinase
Cluster-825.0	-5.0427	1.13E-02	ACO, acnA	Aconitate hydratase
Cluster-495.2276	-4.3041	1.39E-43	argJ	Amino-acid N-acetyltransferase
Cluster-495.7895	-4.0597	1.13E-09	OTC	Ornithine carbamoyltransferase

Cluster-495.6407	-3.5684	6.23E-08	argB	Acetylglutamate kinase
Cluster-495.9112	-3.0144	6.42E-11	pfkA, PFK	6-phosphofructokinase 1
Cluster-495.5005	-2.8583	3.63E-42	argC	N-acetyl-gamma-glutamyl-phosphate reductase
Cluster-495.5217	-2.7963	1.37E-29	TPI, tpiA	Triosephosphate isomerase (TIM)
Cluster-495.6030	-2.7646	4.01E-22	dapB	4-hydroxy-tetrahydrodipicolinate
Cluster-495.4546	-2.7251	3.02E-38	P5CS	Pyrroline carboxylate synthetase
Cluster-495.8773	-2.6734	4.67E-08	PGK	Phosphoglycerate kinase
Cluster-495.4720	-2.6708	7.59E-19	PHGDH	D-3-phosphoglycerate dehydrogenase
Cluster-495.3332	-2.5073	2.29E-19	ADT, PDT	Arogenate dehydratase
Cluster-495.1604	-2.4696	1.42E-08	ALDO	Fructose-bisphosphate aldolase
Cluster-495.9229	-2.4229	1.98E-06	thrA	homoserine dehydrogenase 1
Cluster-495.2017	-2.4122	1.06E-03	ASP5	Aspartate aminotransferase
Cluster-495.3993	-2.3954	7.74E-19	trpG	Anthranilate synthase component
Cluster-495.9065	-2.2275	1.16E-05	cysK	Cysteine synthase A
Cluster-495.4722	-2.0943	3.43E-13	lysA	Diaminopimelate decarboxylase
Cluster-495.6013	-1.9431	7.28E-17	GOT2	Aspartate aminotransferase
Cluster-495.5038	-1.6083	3.40E-17	PK, pyk	Pyruvate kinase
Cluster-495.8045	-1.6067	6.94E-06	GAPDH	Glyceraldehyde 3-phosphate dehydrogenase
Cluster-495.5974	-1.5814	3.82E-15	cysK	Cysteine synthase A
Cluster-495.2271	-1.5651	1.04E-03	gpmB	Probable phosphoglycerate
Cluster-495.3018	-1.5549	4.45E-05	rpiA	Ribose 5-phosphate isomerase A
Cluster-495.2447	-1.5371	2.17E-05	cysE	Serine O-acetyltransferase
Cluster-495.7512	-1.4877	1.89E-04	proC	Pyrroline-5-carboxylate reductase
Cluster-495.4366	-1.4535	7.04E-09	talA, talB	Transaldolase
Cluster-495.8972	-1.4346	1.55E-03	leuA	2-isopropylmalate synthase
Cluster-495.8285	-1.4321	2.60E-02	tktA, tktB	Transketolase

Cluster-495.6710	-1.3187	9.42E-08	IDH1, IDH2	Isocitrate dehydrogenase
Cluster-495.6227	-1.2768	9.08E-09	PK, pyk	Pyruvate kinase
Cluster-495.5492	-1.2187	2.33E-08	ALDO	Fructose-bisphosphate aldolase
Cluster-495.4742	-1.1802	4.04E-11	GAPDH	Glyceraldehyde 3-phosphate dehydrogenase
Cluster-495.5592	-1.1265	5.10E-05	gpmI	2,3-bisphosphoglycerate-independent phosphoglycerate
Cluster-495.2179	-1.1187	8.28E-06	trpC	3-glycerol phosphate synthase
Cluster-495.6190	-1.0435	5.51E-05	cysE	Serine O-acetyltransferase
Cluster-495.4437	-1.0384	9.24E-06	proC	Pyrroline-5-carboxylate reductase
Cluster-495.6855	-1.0211	9.92E-06	trpC	3-glycerol phosphate synthase
Cluster-495.4071	1.3639	1.42E-05	argAB	Amino-acid N-acetyltransferase
Cluster-495.7215	1.4168	6.87E-08	GGAT	Glutamate--glyoxylate aminotransferase
Cluster-495.6465	1.7988	5.96E-22	ilvE	Branched-chain amino acid aminotransferase
Cluster-495.5645	2.2190	1.32E-29	dapF	Diaminopimelate epimerase
**Fatty acid biosynthesis**				
Cluster-495.4487	-4.8270	2.34E-47	fabI	Enoyl-[acyl-carrier-protein] reductase
Cluster-495.3921	-3.6122	2.01E-44	fabD	CoA-acyl carrier protein transacylase
Cluster-495.2280	-3.3685	1.10E-37	fabG	3-oxoacyl-[acyl-carrier-protein] reductase
Cluster-495.3591	-3.0147	1.54E-30	fabF	3-oxoacyl-[acyl-carrier-protein] synthase II
Cluster-495.5787	-2.3116	3.35E-14	fadD	Long-chain acyl-coa synthetase
Cluster-495.5796	-2.2895	4.74E-20	accC	Acetyl-CoA carboxylase

Cluster-495.339	-2.1592	2.17E-04	fabZ	3-hydroxyacyl-[acyl-carrier-protein] synthase
Cluster-495.5391	-1.8857	3.92E-11	fabH	3-oxoacyl-[acyl-carrier-protein] synthase 3
Cluster-495.5077	-1.3533	7.98E-07	FATA	Fatty acyl-ACP thioesterase A
Cluster-495.6352	-1.3369	5.07E-14	fabF	3-oxoacyl-[acyl-carrier-protein] synthase I

Supplementary Table S5. Primers used for qRT-PCR in this study

Primer name	Primer sequence (5′-3′)
Cluster-495.4143 F	GTTCCCCAACGGCGAGGTG
Cluster-495.4143 R	GATGGACACGGGGTTCACG
Cluster-495.6485 F	GGCTGAGCTGAAGGTGAAGGAG
Cluster-495.6485 R	TTGGACCAGTTGGCGTAGGG
Cluster-495.3492 F	AGAACATCTTCCTGGTGGACAGC
Cluster-495.3492 R	TGGGCGTAGGGTAGCTTGTGA
Cluster-495.7973 F	GCACGGCTTTCGAGCATCG
Cluster-495.7973 R	GCCTTGGGAGACAGCGACAGAG
Cluster-495.8475 F	GATAGTGGAGACAGGCCAGAGGC
Cluster-495.8475 R	AGCAGAGCGGGAGCGGAAGT
Cluster-495.7512 F	TGACAGCGAAGACGTGGAGG
Cluster-495.7512 R	GAATGGTTGGATTGGTCGTAAAA
Cluster-495.6811 F	GCGTTCAGCGAGTCCAGAT
Cluster-495.6811 R	CAGCCTCACTTCTATCACAACCA
Cluster-495.165 F	TGCTTGTTGGCTGCTTTGCTG
Cluster-495.165 R	AGCGGATGATGCCGAGGTGT
Cluster-495.5469 F	CCGCACCGAGTGCTTTGAG
Cluster-495.5469 R	CACGCAGTGGATGGGCTTGT
Cluster-495.54 F	ACCTCTGCCACTTCCGCTGTTG
Cluster-495.54 R	TCCTCTAGCACGCCGTCCTCAA
ApActin F	GGCTACGTGGCGCTCGACTA
ApActin R	CGTCATGGAAGGCTGGAACAG
